# Suicide literacy, suicide stigma, and help-seeking attitudes among men in a university setting in Ireland

**DOI:** 10.1093/heapro/daae209

**Published:** 2025-01-24

**Authors:** Shane O’Donnell, Noel Richardson, Aisling McGrath

**Affiliations:** National Centre for Men’s Health, School of Science, South East Technological University (Carlow Campus), Kilkenny Road, Carlow, R93V960, Ireland; National Centre for Men’s Health, School of Science, South East Technological University (Carlow Campus), Kilkenny Road, Carlow, R93V960, Ireland; Centre for Health Behaviour Research, School of Health Sciences, South East Technological University (Waterford Campus), Cork Road, Waterford, X91K0EK, Ireland

**Keywords:** suicide literacy, suicide stigma, help-seeking attitudes, masculine norms, males, university

## Abstract

This study sought to explore the relationship between sociodemographic-, mental health-, knowledge-, attitudinal-, and conformity to masculine norms variables with suicide literacy, suicide stigma, and help-seeking attitudes among men in a university setting (*n* = 471) in Ireland. Multiple linear regression with backward elimination was used to determine the independent variables associated with suicide literacy, suicide stigma, and help-seeking attitudes. Lower suicide literacy was associated with an ethnic minority background, living in a rural community, postgraduate students compared to undergraduate students, no depression symptoms in the past year, decreasing loneliness, greater suicide stigma, more negative help-seeking attitudes, lower resilience, greater conformity to the masculine norm power over women and lower conformity to the masculine norm emotional control. Greater suicide stigma was associated with a non-ethnic minority background, all departments of study compared to health and sports science, lower suicide literacy, more negative help-seeking attitudes, and greater conformity to the masculine norms of power over women, dominance, and heterosexual self-presentation. More negative help-seeking attitudes were associated with no generalized anxiety disorder symptoms in the past year, depression symptoms in the past year, greater suicide risk, lower suicide literacy, greater suicide stigma, greater resilience, and greater conformity to the masculine norms emotional control, self-reliance, violence, and heterosexual self-presentation. Findings highlight a need for gender-responsive psychoeducational programmes to target suicide literacy, suicide stigma, and/or help-seeking attitudes among men in university settings. They also highlight that such initiatives need to be co-produced alongside ethnic minority and rural-dwelling men to ensure they are culturally sensitive and acceptable.

**Contribution to Health Promotion**

- Highlights specific knowledge gaps and stigmatizing attitudes that require attention in relation to suicide prevention among male university students.
- Highlights the need for interventions that reframe and/or challenge masculine norms in order to foster more positive help-seeking attitudes among men in university settings.
- Demonstrates a need for culturally adapted interventions to target men from ethnic minority and rural backgrounds in university settings.

## INTRODUCTION

The prevalence of mental health disorders among university students has increased in the past decade (Lipson et al. 2019). An international survey of 14 000 university students reported that 35% met the criteria for a least one common mental health disorder (Auerbach et al. 2016). More recent meta-analyses have estimated pooled prevalence rates of 25% for depression (Sheldon et al. 2021) and 39.7% for non-specific anxiety respectively (Ahmed et al. 2023). University students are also considered to be vulnerable to suicidal behaviour with estimated pooled lifetime prevalence rates of 22.4% for suicidal ideation, 6.1% for suicide plans, and 3.2% for suicide attempt (Mortier et al. 2018). While suicide rates tend to rise with increasing age (Naghavi 2019), suicide remains the fourth leading cause of death among young people aged 15–29 years old (WHO 2021). Although anxiety, depression, and suicidal behaviour are more common among female university students (Mortier et al. 2018; Liu et al. 2019), male university students are more likely to die by suicide (Gunnell et al. 2020). This phenomenon has been coined the ‘gender paradox’ of suicide (Canetto and Sakinofsky 2010). Various explanations have been proffered to account for this gendered aspect of suicide behavior (Sex and gender are distinct yet interconnected concepts. In this context, sex differences relate to the biological distinction between male and female (Giddens 2009, p. 601), with the terms ‘males’/‘young males’ being used to distinguish categorical differences in mental health outcomes, health behaviours, and suicide behaviours from ‘females’/‘young females’. Gender, on the other hand, encompasses the social, cultural, and psychological roles, behaviours, and identities that a society associates with being masculine or feminine (Connell 2005). Whilst sex is often categorized as binary, gender is diverse and can transcend traditional boundaries. The terms ‘men’ and ‘young men’ are used therefore when describing gendered aspects of mental health and suicide behaviour.), such as men’s use of more lethal methods (Conner et al. 2019), maladaptive emotional regulation and coping strategies (Bennett et al. 2023), and men’s overuse of alcohol and drugs (Richardson et al. 2021). However, one factor that has gained significant scholarly attention is men’s reticence to seek professional help (Clearly 2017; O’Donnell and Richardson 2020). Young men are among the least likely of any demographic group to seek help from a mental health professional (Rickwood et al. 2005; Smith et al. 2024). While previous studies have demonstrated that 16%–36% of university students with a mental health problem have accessed treatment, female students are two to four times more likely to access treatment than male students (McLafferty et al. 2017; Hubbard et al. 2018; Seehuus et al. 2021).

Mental health literacy was originally conceptualized as knowledge about mental health disorders that aids in their recognition, management, and/or prevention (Jorm 2000) while stigma refers to negative attitudes or beliefs towards an individual or group based on particular characteristics (Corrigan et al. 2016). However, more recent work by Kutcher and colleagues has expanded on this definition to highlight the role of mental health literacy in decreasing mental health stigma and enhancing help-seeking efficacy, in addition to understanding mental health disorders, their treatment, and how to obtain and maintain positive mental health (Kutcher et al. 2016). Indeed, mental health literacy and stigma have been highlighted as two of the most common and modifiable psychosocial barriers in relation to help-seeking among young people (Radez et al. 2021). Limited knowledge about suicide (i.e. suicide literacy) and stigmatizing attitudes towards people experiencing suicidality (i.e. suicide stigma) are also reported to represent significant barriers to help-seeking for suicidality (Calear et al. 2022; Al-Shannaq and Aldalaykeh 2023). Poor awareness of the signs and symptoms of suicide can result in an individual not recognizing the severity of suicide risk and/or underestimating the importance of seeking help (Calear et al. 2022). Similarly, individuals with pronounced personal stigma may refrain from help-seeking to avoid contact or association with a stigmatized group (Schnyder et al. 2017).

It is well-established that male university students have significantly poorer mental health literacy and higher mental health stigma compared to female students (Clough et al. 2019; Eisenberg et al. 2009; Gorczynski et al. 2017; Rafal et al. 2018; Pompeo-Fargnoli 2022). However, there is mixed evidence in relation to sex differences in suicide literacy and suicide stigma among university students. A number of studies have reported significantly lower suicide literacy and higher suicide stigma among male university students compared to female university students (Batterham et al. 2013; Rivera-Segarra et al. 2018; Williams and Witte 2018; Arafat et al. 2022). Conversely, other studies have reported no significant differences (Chan et al. 2014; Han et al. 2017; Öztürk and Akın 2018). Notwithstanding the mixed evidence regarding potential sex differences in these variables for this population, no study to date has explored the factors associated with suicide literacy and suicide stigma among men in university settings. This represents a significant gap in the literature considering that male students are less likely than female students to access treatment, which may in part be associated with poorer suicide literacy and higher suicide stigma. A number of studies have highlighted a significant relationship between lower university year, non-health or psychology-related study, and an ethnic minority background with poorer suicide literacy and higher suicide stigma (Batterham et al. 2013; Chan et al. 2014; Han et al. 2017; Öztürk and Akın 2018; Rivera-Segarra et al. 2018; Williams and Witte 2018; Arafat et al. 2022; Žilinskas and Lesinskienė 2023). The same studies reported mixed evidence in relation to the effect of age, relationship status, religious status, generalized anxiety disorder (GAD), depression, and a history of suicidality and/or exposure to suicide. Whether these sociodemographic, knowledge, attitudinal, and/or mental health variables are associated with suicide literacy or suicide stigma among men in university settings remains to be explored. Indeed, there have been calls for a greater understanding of the factors associated with suicide literacy and suicide stigma among men more generally (Oliffe et al. 2016a, 2016b).

Conformity to masculinity norms within university settings has been attributed to men’s reluctance to seek help for mental health problems (Nam et al. 2010; Siedler et al. 2016). Conceptualizations of masculinity have moved away from the notion of a singular and static ‘masculinity’, recognizing a plurality of ‘masculinities’ that are fluid, relational, and co-existent (Connell and Messerschmidt 2005). Central to these social constructionist theories is that adherence to ‘hegemonic’ masculine ideologies influences men’s health practices and illness experiences. Hegemonic masculinities hold their position of power through cultural beliefs in an idealized ‘masculine standard’ that is most often characterized by a desire for power, strength, emotional control, aggression, self-reliance, independence, and avoidance of negative emotions (Möller-Leimkühler 2003; Payne et al. 2008; Pirkis et al. 2017). Men in university settings who more strongly endorse conformity to such masculine norms may choose not to seek help on the basis of help-seeking being seen as incongruent with cultural ideals of hegemonic masculinities (Tang et al. 2014; Sagar-Ouriaghli et al. 2020). Indeed, conformity to masculine norms tends to be most strongly endorsed by younger men (Rice et al. 2011) and, in the context of university settings, has been reported to account for over a quarter of the variance in men’s intention to seek help (Smith *et al.*, 2008). While some masculine norms may be adaptive and facilitate economic and social benefit to men in university settings (Iwamoto et al. 2018), conformity to the masculine norms of self-reliance and emotional control are particularly associated with lower intentions to seek help among men in these settings (Davies et al. 2000; McDermott et al. 2018; Sagar-Ouriaghli et al. 2020). It is also possible that different masculine norms may have a relationship with suicide literacy and suicide stigma, but this remains to be explored. Previous studies have highlighted a relationship between conformity to masculine norms and both mental health literacy and stigma (Nam et al. 2010; Steinfeldt and Steinfeldt 2012; Miles et al. 2020), as well as health literacy more generally (Milner et al. 2019). However, no study to date has explored whether conformity to specific masculine norms is associated with suicide literacy and/or suicide stigma. Indeed, focussing solely on composite measures of masculine norms can mask important insights into specific dimensions of masculinities that may be uniquely associated with suicide literacy, suicide stigma, and/or help-seeking attitudes (Gerdes and Levant 2018). Identifying these relationships may assist in the development of more targeted gender-responsive campaigns and interventions to improve suicide literacy, reduce suicide stigma, and foster more positive help-seeking attitudes among men in university settings. Therefore, the aim of this exploratory study was to explore the relationship between sociodemographic-, mental health-, knowledge-, attitudinal-, and conformity to masculine norm variables with suicide literacy, suicide stigma, and help-seeking attitudes among men in a university setting in Ireland.

## METHODS

### Study design

A cross-sectional study was conducted at a Technological University in Ireland between 6 November and 19 November 2023. Ethical approval was granted by the Southeast Technological University Carlow Ethics Committee (Reference No. 380). There were approximately 9000 male students enrolled in the university at the time of data collection. Individuals were eligible to participate if they identified as male, were ≥18 years old, and were enrolled as a full-time student in the university.

### Data collection procedures

Data were collected online via a self-report survey using the Qualtrics software. The timing of the survey sought to capitalize on a range of events that were scheduled to celebrate International Men’s Day (IMD) on the 19 November 2023. A member of the research team distributed recruitment emails to all male students in the university 1 week prior to IMD, 2 days prior to IMD, the day of IMD, and 2 days after IMD. This email contained a study information sheet and a link to the survey. Posters containing QR codes with links to the information sheet and the survey were also displayed in social spaces across the university. Finally, members of the research team also recruited participants via health promotion stands that were positioned across university campuses during IMD. Participants were given health promotion leaflets, study information sheets and were encouraged to complete the survey online in their own time. All students completed an online informed consent prior to participation. On completion of the survey, all students were provided with information on how to access mental health services in the university and in the local community. Students who reported experiencing suicidality were provided additional information on available services via pop-up messages that were embedded into the online survey.

### Dependant variables

#### Suicide literacy

The 12-item Literacy of Suicide Scale Short-Form (LOSS-SF) was used to assess suicide literacy. This measure was previously validated in a university-based sample (Calear et al. 2022). Each item on the LOSS-SF elicits a response of ‘true’, ‘false’, or ‘I don’t know’. Correct responses are assigned a score of one and incorrect or ‘I don’t know’ responses are assigned a score of zero. Total scores ranged from 0 to 12 and were calculated by summing the number of correct responses. Higher LOSS-SF scores are indicative of higher suicide literacy.

#### Suicide stigma

The 16-item Stigma of Suicide Scale Short-Form (SOSS-SF) assesses attitudes towards suicide decedents and has been previously validated in a university sample (Batterham et al. 2013). The SOSS-SF consists of one- or two-word descriptors for suicide decedents and responses range from strongly disagree (1) to strongly agree (5). Previous factor analysis showed three subscales that assess: suicide stigma (8 items, e.g. weak, embarrassment); attribution of suicide to isolation/depression (4 items, e.g. disconnected, lonely); and glorification or normalization of suicide (4 items such as dedicated, strong). Only the suicide stigma subscale was used in this study. The subscale is scored as the mean response to items loading on each subscale ranging from 1 to 5.

#### Help-seeking attitudes

The 10-item Attitudes towards Seeking Professional Psychological Help—Short-Form (ATSPPH-SF) was used to assess help-seeking attitudes. The ATSPPH-SF has good psychometric properties and a strong correlation with the original 29-item scale (Fischer and Farina 1995). Items are rated on a four-point Likert scale ranging from disagree (0) to agree (3) where 2, 4, 8, 9, and 10 are reversed scored. Responses are summed to give a total score ranging from 0 to 30 with higher scores indicating more positive attitudes towards help-seeking.

### Independent variables

#### Conformity to masculine norms

The Conformity to Masculine Norms Inventory-22 (CMNI-22) was used to assess conformity to masculine norms. The CMNI-22 is an abbreviated version of the original 94-item scale that uses the two highest loading statements from each of the eleven factors. CMNI-22 has a correlation of 0.92 with the original scale (Mahalik et al. 2003) and has demonstrated good psychometric properties (King et al. 2020). The 11 factors form subscales that include: (i) importance of winning; (ii) importance of emotional control; (iii) endorsement of risk-taking; (iv) endorsement of violence; (v) salience of power over women; (vi) importance of being dominant; (vii) salience of playboy status; (viii) importance of self-reliance; (ix) importance of heterosexual self-presentation; (x) importance of social status; and (xi) primacy of work. Response options range from strongly disagree (0) to strongly agree (3) for each item. The two items for each subscale are summed to give an overall subscale score ranging from 0 to 6 where higher scores indicate greater conformity to each masculine norm.

#### Suicide risk

The four-item Suicidal Behaviour Questionnaire Revised (SBQ-R) was used to assess lifetime suicidal behaviour, frequency of suicidal ideation in the past year, the threat of a suicide attempt, and the likelihood of suicidal behaviour in the future. Responses are summed to give a total suicide risk score of 3–18 where higher scores indicate greater suicide risk. A cut-off score of ≥7 is indicative of suicide risk (Osman et al. 2001).

#### Loneliness

The UCLA Three-Item Loneliness Scale (UCLA-3) was used to assess loneliness and has previously shown good psychometric properties (Russell 1996). Responses across the three items range from hardly ever (1) to often (3) and are summed to give a total score ranging from 3 to 9 where higher scores indicate a higher degree of loneliness. A cut-off of ≥6 can be used as a classification of ‘lonely’ (Steptoe et al. 2013).

#### Generalized anxiety disorder and depression

Generalized anxiety disorder (GAD) and depression were assessed using single dichotomous items that asked about the presence of symptoms for each in the past year that were based on the Australian Health Survey (Pirkis et al. 2017).

#### Resilience

The six-item Brief Resilience Scale (BRS) was used to measure the ability to bounce back or recover from stress (Smith et al. 2008). The BRS is measured on a four-point Likert scale with items ranging from strongly disagree (1) to strongly agree (5). The BRS is scored by reverse coding items 2, 4, and 6, and then calculating the mean of the six items. The overall average score was used with higher scores indicated a greater degree of resilience.

#### Spirituality

A single item was used to assess the importance of spirituality which was measured on a four-point Likert scale where higher scores indicate a greater importance of spirituality in life.

#### Sociodemographic characteristics

Seven sociodemographic questions were included: age; ethnicity (White Irish or other White background; Black Irish or other Black background; Asian Irish or other Asian background; Arabic Irish or other Arabic background; other); sexual orientation (gay; bisexual; heterosexual; other); relationship status (in a committed relationship; not in a committed relationship); geographical location (rural location <1500 people; urban location > 1500 people); year of study (undergraduate year one; undergraduate year two; undergraduate year three; undergraduate year four; postgraduate); department of study (engineering and built environment; business, humanities, and education; computing; health and sports science; science); and suicide bereavement (yes/no). Geographical location, ethnicity, and sexual orientation questions were taken from the Irish Census survey. The latter two items were dichotomized into binary variables (0 = non-ethnic minority and 1 = ethnic minority; 0 = heterosexual, 1 = gay/bisexual/other).

### Data analysis

All analyses were conducted in STATA 18. Cronbach’s *α* was computed as a measure of internal reliability for SOSS-SF Stigma (*α*: 0.73); ATSPPH-SF (*α*: 0.72); CMNI-22 (*α:* 0.70); UCLA-3 (*α:* 0.83); BRS (*α*: 0.76); and SBQR-SF (*α*: 0.81). Internal consistency was not computed for the LOSS-SF due to items on the scale carrying true or false answers. Multiple linear regression with backward elimination was used to determine the factors associated with suicide literacy, suicide stigma, and help-seeking attitudes respectively. Backward elimination starts with a global model that includes all candidate independent variables. The variable with the highest *P*-value is removed and the model is re-estimated (Heinze et al. 2018). The process is repeated until all remaining variables have a *P*-value below a pre-specified significance threshold. This backward elimination process was conducted for each of the three dependent variables separately with a significance threshold of *P* < .05. Assumptions of normality, linearity, multicollinearity, and homoscedasticity were met in all three regression models.

## RESULTS

A total of 471 male university students participated in this study. The median age of participants was 20 years old (IQ range: 19–22 years old) and was largely from a non-ethnic minority background (81.5%), heterosexual (80.2%), and living in a rural community (61.9%). Notably, 31.3% had experienced a suicide bereavement, 64.1% were classified as lonely (score of ≥6), 52.8% and 40% reported symptoms of generalized anxiety disorder and depression in the past year respectively, and 42.5% met the criteria for suicide risk (score of ≥7). Participant characteristics are reported in Table 1.

**Table 1.** Participant characteristics

	Number	%
Total sample	471	100
Ethnicity		
Ethnic minority	87	18.5
Non-ethnic minority	384	81.5
Sexual orientation		
Gay/bisexual/other	90	19.2
Heterosexual	379	80.8
Relationship status		
Not in a committed relationship	187	40.2
In a committed relationship	278	59.8
Geographical location		
Urban	288	61.9
Rural	177	38.1
Department of study		
Engineering & built environment	129	28.2
Business, humanities & education	157	34.4
Computing	63	13.8
Health & sport science	61	13.3
Science	47	10.3
Stage of study		
Undergraduate, Year 1	170	37.9
Undergraduate, Year 2	115	25.6
Undergraduate, Year 3	60	13.4
Undergraduate, Year 4	66	14.7
Postgraduate	38	8.5
Suicide bereavement		
No	320	68.7
Yes	146	31.3
GAD symptoms (past year)		
No	216	47.2
Yes	242	52.8
Depression symptoms (past year)		
No	275	60
Yes	183	40
UCLA-3-item Loneliness Scale		
Not lonely	164	35.9
Lonely	293	64.1
SBQR		
No suicide risk	271	57.5
Suicide risk	200	42.5

### Suicide literacy

The mean score for suicide literacy was 7.1 (SD 3.1), equivalent to 59.2% correct responses. Participants tended to have more difficulty with correctly identifying signs/symptoms (47.5%) and causes/nature (58.9%), and less difficulty with correctly identifying risk factors (65.4%) and treatment and prevention items (68.2%). Rates of correct responses to the LOSS-SF items are shown in Supplementary Table 1. The multiple linear regression model for suicide literacy was significant (F (10, 406) = 11.5, *P* < .001) and explained 22.0% of the variance in suicide literacy. Lower suicide literacy was associated with an ethnic minority background (*P* = .022), living in a rural community (*P* = .044), postgraduate students compared to all undergraduate years (*P* = .035), no depression symptoms in the past year (*P* = .001), lower levels of loneliness (*P* = .008), greater suicide stigma (*P* = .039), more negative help-seeking attitudes (*P* = .001), lower resilience (*P* = .007), greater conformity to the masculine norm power over women (*P* = 0016) and lower conformity to the masculine norm emotional control (*P* = .045). See Table 2 for more details.

**Table 2.** Multiple linear regression for suicide literacy

	LOSS-SF total		
	Coef.	*P*-value	95% CI
Ethnicity			
Ethnic minority	Ref	**–**	**–**
Non-ethnic minority	0.87	0.022	0.04, 0.18
Geographical location			
Urban	Ref	**–**	**–**
Rural	–0.56	0.044	–1.11, –0.13
Stage of study			
Undergraduate, years 1–4	Ref	–	**–**
Postgraduate	–1.10	0.035	–2.13, –0.08
Depression symptoms (past year)			
No	Ref	**–**	**–**
Yes	1.03	0.001	0.44, 1.63
UCLA 3-item loneliness scale	0.21	0.008	0.05, 0.36
SOSS-SF stigma	–0.37	0.039	–0.72, –0.18
ATSPPH—SF	0.11	0.001	0.04, 0.18
Brief resilience scale	0.49	0.007	0.13, 0.85
CMNI-22 emotional control	0.18	0.045	0.01, 0.36
CMNI-22 power over women	–0.31	0.016	–0.56, –0.06

### Suicide stigma

The average suicide stigma score was 2.0 (SD 0.9). Few participants endorsed overtly stigmatizing items, although 16%–17% of the sample endorsed ‘irresponsible’, ‘stupid’, or ‘cowardly’ as descriptors of suicide decedents. Responses to the SOSS-SF Stigma are shown in Supplementary Table 2. The multiple linear regression model for suicide stigma was significant (F (7, 409) = 34.4, *P* < .001) and explained 37.0% of the variance in suicide stigma. Greater suicide stigma was associated with a non-ethnic minority background (*P* = .021), all departments of study compared to health and sports science (*P* = .022), lower suicide literacy (*P* = .018), more negative attitudes towards help-seeking (*P* < .001); and greater conformity to the masculine norms power over women (*P* < .001), dominance (*P* = .003), and heterosexual self-presentation (*P* < .001). See Table 3 for more details.

**Table 3.** Multiple linear regression for suicide stigma

	SOSS-SF: Stigma		
	Coef.	*P*-value	95% CI
Ethnicity			
Ethnic minority	Ref	**–**	–
Non-ethnic minority	–0.22	0.021	–0.41, –0.03
Department of study			
Engineering & built environment; business, humanities & education; computing; and science	Ref	–	–
Health & sports science	–0.24	0.022	–0.45, –0.04
LOSS-SF	–0.03	0.018	–0.05, –0.02
ATSPPH-SF	–0.03	<0.001	–0.05, –0.01
CMNI-22 power over women	0.17	<0.001	0.11, 0.24
CMNI-22 dominance	0.09	0.003	0.03, 0.16
CMNI-22 heterosexual self-presentation	0.12	<0.001	0.08, 0.17

### Help-seeking attitudes

The mean score for help-seeking attitudes was 15.7 (SD 4.5). The multiple linear regression model for help-seeking attitudes was significant (F 10, 406) = 24.4, *P* < .001) and explained 37.5% of the variance in help-seeking attitudes. More negative help-seeking attitudes were associated with no GAD symptoms in past year (*P* = .011), depression symptoms in past year (*P* = .020), greater suicide risk (*P* = .016), lower suicide literacy (*P* = .001), greater suicide stigma (*P* < .001), greater resilience (*P* < .001), and greater conformity to the masculine norms emotional control (*P* < .001), violence (*P* = .013), self-reliance (*P* = .022), and heterosexual self-presentation (*P* = .002). See Table 4 for more details.

**Table 4.** Multiple linear regression for help-seeking attitudes

	ATSPPH-total		
	Coef.	*P*-value	95% CI
GAD symptoms (past year)			
No	Ref	**–**	–
Yes	1.00	0.011	0.23, 1.78
Depression symptoms (past year)			
No	Ref	**–**	–
Yes	–1.03	0.020	–1.89, –0.16
SBQR-total	–0.15	0.016	–0.27, –0.03
LOSS-SF	0.22	0.001	0.09, 0.35
SOSS-SF stigma	–1.05	<0.001	–1.52, –0.58
Brief resilience scale	–1.16	<0.001	–1.65, –0.66
CMNI-22 emotional control	–0.84	<0.001	–1.08, –0.60
CMNI-22 violence	–0.33	0.013	–0.58, –0.07
CMNI-22 self-reliance	–0.32	0.022	–0.60, –0.05
CMNI-22 heterosexual self-presentation	–0.38	0.002	–0.63, –0.14

## DISCUSSION

This study sought to explore the multifaceted factors associated with suicide literacy, suicide stigma, and help-seeking attitudes among men in university settings. Findings underscore the nuanced interplay of sociodemographic characteristics, mental health indicators, and masculine norms that shape men’s knowledge and attitudes with regard to suicide and help-seeking in university settings. The mean suicide literacy score (59% correct) in this sample is slightly less than the median score of 63% reported among mixed-sex university samples (Calear et al. 2022). The mean suicide stigma score of 2.0 is in line with previous studies (Chan et al. 2014; Han et al. 2018). While this may indicate a greater need to improve suicide literacy among male students, it should be acknowledged that no data were collected on females, and therefore it is not possible to make robust comparisons. More research on suicide literacy and suicide stigma among university students with sex-disaggregated data are needed to make more concrete conclusions. However, findings highlight notable variations in suicide literacy and suicide stigma scores across different demographic and psychosocial factors among men in university settings. Participants appeared to have more difficulty answering questions related to identifying the signs and symptoms of suicide as well as its causes and nature. In particular, only 39% of students correctly identified that people who want to attempt suicide can change their minds quickly while only 37% correctly identified that the statement ‘people who talk about suicide rarely kill themselves’ was false.

Findings in this study highlight that greater conformity to the masculine norm power over women was linked to lower suicide literacy while greater conformity to emotional control was associated with greater suicide literacy. Additionally, greater conformity to the masculine norms of power over women, dominance, and heterosexual self-presentation were predictors of greater suicide stigma. These norms collectively reflect hegemonic attitudes that legitimize men’s dominant position in society and justify the subordination of women and marginalized groups of men (Connell and Messerschmidt 2005). It is likely that men who more strongly endorse these hegemonic attitudes have a greater propensity to hold more stigmatizing attitudes towards people experiencing suicidality and to possess a desire for social distance from such groups (Schnyder et al. 2017), on the basis that suicidality is a sign of weakness that is incongruent with hegemonic attitudes. Moreover, conformity to hegemonic masculinities has also been found to undermine men’s ability to understand, process, and recognize psychological distress (Bennett et al. 2023), while previous research has also reported that greater endorsement of the masculine norm power over women is significantly associated with devaluing intellectual engagement (Marrs 2013). Therefore, this may limit some men’s ability to recognize suicide signs/symptoms and may reduce their willingness to engage in extant campaigns and psychoeducational programmes thus resulting in lower suicide literacy. These findings highlight that hegemonic attitudes not only represent social injustices to women and other groups of men (Connell and Messerschmidt 2005) but also have deleterious consequences for men who embrace them. This aligns with a meta-analysis that reported power over women and self-reliance as particularly salient masculine norms that have a negative relationship with mental health outcomes among men (Wong et al. 2017). Greater conformity to the masculine norm of emotional control has previously been noted as a barrier to help-seeking and a risk factor for suicidal ideation (McDermott et al. 2018). Perhaps men with greater conformity to the masculine norm of emotional control are more likely to experience mental ill-health as a result of not seeking timely support, and thus be more acutely aware of the signs, causes, and nature of suicide (i.e. greater suicide literacy). Lastly, greater conformity to the masculine norms of self-reliance, emotional control, violence, and heterosexual self-presentation were associated with more negative help-seeking attitudes. The relationship between more negative help-seeking attitudes and conformity to the masculine norms of self-reliance and emotional control is well-established in the field of men’s health (Wong et al. 2017; McDermott et al. 2018; Mahalik and DiBianca 2021). Previous research has highlighted a moderate relationship between violence and informal help-seeking intentions among college students (McDermott et al. 2018) and an inverse relationship between violence, self-acceptance, and talking to a mental health professional about depression (Gerdes and Levant 2018). This suggests that conformity to violence may in some way, be a mechanism to deny vulnerability, and aligns with the notion that men may cope with distress through more ‘male-acceptable’ outlets such as aggression and irritability (O’Donnell and Richardson 2018). Similarly, men who more strongly adhere to the masculine norm of heterosexual self-presentation may avoid help-seeking because it represents a sign of weakness that is incongruent with hegemonic ideals.

This study highlighted that men from ethnic minority backgrounds in university settings have significantly poorer suicide literacy and higher suicide stigma compared to those from non-ethnic minority backgrounds which is consistent with previous studies (Chan et al. 2014; Calear et al. 2022). This could reflect a lower acceptability of suicide in some cultures as well as cultural preferences for conservatism and privacy regarding personal matters (Al-Shannaq and Aldalaykeh 2023). Indeed, previous studies conducted among university students in Asian countries such as Bangladesh and Turkey scored considerably lower on overall suicide literacy and higher on suicide stigma (Öztürk and Akın 2018; Arafat et al. 2022) compared to studies conducted in Australia or Europe (Han et al. 2018; Žilinskas and Lesinskienė 2023). Moreover, those living in rural areas had significantly lower suicide literacy and higher suicide stigma compared to those living in urban areas. This aligns with previous studies that postulated poorer suicide literacy and higher suicide stigma in rural communities to be a result of limited mental health services, mental health education programmes, and suicide prevention resources as well as small social networks that restrict anonymity and create a sense of uneasiness around confiding in others (Judd et al. 2006; Batterham et al. 2013; Kennedy et al. 2018). However, more research relating to suicide literacy and suicide stigma that accounts for geographical location is needed before recommendations can be made in this regard. In terms of mental health variables, this study reported that poorer suicide literacy was associated with depression symptoms in the past year and lower levels of loneliness. This is in contrast to previous studies (Batterham et al. 2013). Perhaps those with past depression symptoms and experiences of loneliness are more familiar with the signs, symptoms, causes, and treatment of mental ill-health, and subsequently suicide, as a result of their own personal experience, however further investigation is required. Similarly, more negative help-seeking attitudes were associated with depression symptoms in the past year and increasing suicide risk which aligns with previous research (Michelmore and Hindley 2012; Rastogi et al. 2024). Interestingly, GAD symptoms in the past year were associated with more positive help-seeking attitudes. Depression and suicidality are often characterized by hopelessness, worthlessness, and perceived burdensomeness (Van Orden et al. 2006; American Psychiatric Association 2013), which may make the prospect of seeking help feel overwhelming or result in a perception that seeking help won’t lead to improvement. Conversely, GAD is often characterized by feelings of persistent worry, hypervigilance, and a desire for certainty (Newman et al. 2013), which may be more conducive to more positive help-seeking attitudes. Alternatively, these findings may reflect differences in perceived- and self-stigma associated with GAD, depression, and suicidality among men in university settings. However, due to the cross-sectional nature of this study, it is not possible to determine the causality or directionality of the relationship between GAD, depression, and suicide risk with help-seeking attitudes. Therefore, it is not possible to determine if help-seeking attitudes are a cause or a symptom of GAD, depression, and/or suicide risk. Similarly, the directionality of the relationship between resilience, suicide literacy, and more negative help-seeking attitudes is unclear and requires further investigation. Lastly, suicide literacy, suicide stigma, and help-seeking attitudes were all significantly associated with each other. This aligns with previous studies and reviews that highlight an inverse relationship between these variables among university students (Rivera-Segarra et al. 2018; Williams and Witte 2018; Calear et al. 2022).

### Practical implications and future directions

Findings from this study highlight that men in university settings appear to have the most difficulty identifying the signs and symptoms of suicide as well as its causes and nature. This may reflect specific knowledge gaps relating to the seriousness of someone talking about suicide and the importance of intervention to prevent suicide. This could be targeted through university-wide awareness campaigns and/or gatekeeper training programmes (Burnette et al. 2015). The findings with regard to the relationship between hegemonic masculine norms (e.g. dominance, heterosexual self-presentation, power over women, and violence) and suicide literacy, suicide stigma, and help-seeking attitudes warrant concern, given the recent growth in the ‘manosphere’—a network of online communities within which male supremacist and anti-women discourses flourish—of which young men are particularly susceptible (Verma and Khurana 2023). This copper fastens the need for interventions that engender progressive, healthy forms of masculinities among young men and that promote more gender-equal and equitable attitudes. Such gender-transformative programmes may have additional benefits in addressing stigmatizing attitudes and knowledge gaps in relation to mental health and suicide (Wilson et al. 2022). However, such interventions should work with, and not against, cultural ideals of masculinities to ensure their overall acceptability (O’Donnell et al. 2023). The positive masculinities framework developed by Wilson et al. (2022) would be particularly useful in this regard. Nonetheless, campaigns and interventions that aim to encourage more positive help-seeking attitudes among men in university settings should consider, more broadly, how they can address these hegemonic masculine attitudes in addition to reframing other norms such as self-reliance and emotional control. However, it is crucial that such interventions are underpinned by appropriate theories relating to stigma-reduction and help-seeking such as the theory of planned behaviour (Roche et al. 2024; Sweeney et al. 2024). While these findings begin to explain the relationship between conformity to masculine norms and the dependant outcomes of interest, further research is needed to identify if these relationships exist within other samples of men in university settings and within men more generally.

Findings in this study that relate to lower suicide literacy and greater suicide stigma among men from an ethnic minority background or men living in rural communities highlight the need for comprehensive interventions and targeted strategies that account for these contextual factors (Handley et al. 2021; McKay and Meza 2024). Moreover, it indicates the need for co-produced psychoeducational programmes that are culturally adapted to suit the specific needs of ethnically diverse, and rural-dwelling men in university settings, which has implications for wider suicide prevention programmes targeting other marginalized groups of men considered ‘at risk’ of suicide (Richardson et al. 2023). Lastly, study findings reaffirm the role of knowledge in combatting suicide stigma among men in university settings, and the potential role of stigma-reduction in fostering more positive help-seeking attitudes. However, future research would benefit from structural equation models that explore the mediating and moderating relationships between conformity to masculine norms, suicide literacy, suicide stigma, and help-seeking attitudes.

### Limitations

This study has a number of limitations that should be taken into consideration. The cross-sectional design of the study prevents the assessment of causality, meaning it is not possible to determine the directionality of the relationships observed between the independent variables and the primary outcomes. This limitation is important as it restricts the ability to infer mechanisms or establish temporal precedence, requiring caution in drawing causal conclusions. This study was conducted among male students from a single university in Ireland, with limited diversity in terms of ethnicity and sexuality. This lack of representativeness constrains the generalisability of the findings to the broader population of men in university settings. Future studies with more representative and diverse samples would enhance the applicability of these findings. There was a low response rate to the survey relative to the number of male students that were contacted. This self-selection could mean that individuals for whom suicide resonated more strongly may have been more likely to participate, introducing a non-response bias. Consequently, the sample may disproportionately reflect individuals at higher suicide risk, potentially inflating scores on some outcome measures and affecting the representativeness of the observed associations. Another limitation concerns the use of single-item measures to assess GAD, depression, and the importance of spirituality. Single-item measures can lack precision, reliability, and construct validity compared to validated scales (Allen et al. 2022) which may result in measurement error and less robust estimates of associations involving these variables. Interpretations of findings related to these measures should therefore be made cautiously. Nonetheless, these single-item measures can serve as proxies measures (Pirkis et al. 2017) and future use of validated scales to explore the relationships between GAD and depression and the dependent outcomes is recommended. Although not insignificant, the proportion of variance explained by some of the regression models was relatively small, suggesting that other variables that are associated with the study outcomes were not accounted for in this study. These limitations highlight the exploratory nature of this study. Future research should seek to build on the findings of this study to identify additional variables of interest to provide a more comprehensive understanding of the factors associated with suicide literacy, suicide stigma, and help-seeking attitudes among men in university settings.

## CONCLUSION

This study provides valuable insights into the complex interplay of sociodemographic-, mental health-, knowledge and attitudinal-, and conformity to masculine norms variables with suicide literacy, suicide stigma, and help-seeking attitudes among men in a university setting. The findings highlight several critical gaps and challenges, including the need to address low suicide literacy, particularly the recognition of signs, symptoms, and causes of suicide. The influence of masculine norms, such as power over women, heterosexual self-presentation, dominance, violence, self-reliance, and emotional control, highlights the significant role of gendered constructs in shaping knowledge and attitudes toward suicide and help-seeking behaviours. Additionally, men from ethnic minority backgrounds and rural communities were found to experience disproportionately lower suicide literacy and higher stigma, further emphasizing the necessity for targeted and culturally sensitive interventions. Interventions aimed at improving suicide literacy and reducing stigma should consider the nuanced sociocultural factors that influence these outcomes. Gender-transformative programmes that promote healthy masculinities, alongside psychoeducational campaigns tailored to diverse and underserved groups, may hold promise in improving suicide literacy and fostering more positive attitudes towards suicide and help-seeking attitudes. While the study’s exploratory nature and methodological limitations temper the generalizability of its findings, they also pave the way for future research to build upon these associations, ultimately guiding the development of more effective suicide prevention strategies for this population.

## Supplementary Material

daae209_suppl_Supplementary_Tables_1-2

## Data Availability

The datasets used and/or analysed during the current study are available from the corresponding author upon reasonable request.
