# Supplementary material for: Suicide literacy, suicide stigma, and help-seeking attitudes among men in a university setting in Ireland
Source: Health Promot Int. 2025 Jan 24;40(1):daae209. doi: 10.1093/heapro/daae209 (PMC11759269; doi:10.1093/heapro/daae209)
Supplement: daae209_suppl_Supplementary_Tables_1-2 [file daae209_suppl_supplementary_tables_1-2.docx]

***Supplementary File 1***

**Supplementary Table 1: Correct Responses to Items from Literacy of Suicide Scale**

| **Item** | **Correct, %** |
| --- | --- |
| People who have thoughts about suicide should not tell others about it (F) | 68.1 |
| Seeing a psychiatrist or psychologist can help prevent someone from suicide (T) | 68.6 |
| Most people who suicide are psychotic (F) | 65.0 |
| Talking about suicide always increases the risk of suicide (F) | 61.4 |
| A suicidal person will always be suicidal and entertain thoughts of suicide (F) | 58.3 |
| Not all people who attempt suicide plan their attempt in advance (T) | 66.3 |
| Very few people have thoughts about suicide (F) | 63.3 |
| If assessed by a psychiatrist, everyone who kills themselves would be diagnosed as depressed (F) | 52.8 |
| Men are more likely to die by suicide than women (T) | 72.7 |
| People who talk about suicide rarely kill themselves (F) | 37.6 |
| People who want to attempt suicide can change their mind quickly (T) | 39.0 |
| There is a strong relationship between alcoholism and suicide (T) | 58.8 |

**Supplementary Table 2: Responses to Items from SOSS-SF Stigma**

| **Item** | **Agree/strongly agree, %** | **Mean (SD) rating** |
| --- | --- | --- |
| Pathetic | 9.8 | 2.03 (1.07) |
| Shallow | 8.4 | 1.99(1.03) |
| Immoral | 7.5 | 1.98 (1.01) |
| An embarrassment | 5.3 | 1.81 (0.94) |
| Irresponsible | 17.5 | 2.16 (1.18) |
| Stupid | 16.4 | 2.18(1.20) |
| Cowardly | 16.2 | 2.12 (1.18) |
| Vengeful | 4.7 | 1.93(0.98) |
